# Dr. Sherwen, on Digitalis

**Published:** 1800-04

**Authors:** John Sherwen

**Affiliations:** Enfield


### *To the Editors of the Medical and Phyfical Journal.*

GENTLEMEN,

IN the laft Number of your very refpectable Journal, which affords a ready and convenient medium of communication to medical men, I recognifed, with much pleafure, a fignature affixed to an ingenious paper on the Phymofis, promifing other communications, to which I look forward with much fatisfaction and expectation.—After a lapfe of thirty years, to meet with the name of a gentleman whom I had known and refpected in the fame fervice with myfelf in the Eaft Indies, added much to the gratification which the perufal of your Journal always affords.

I obferve, in the fame Number, but with fomewhat lefs of the agreeable, that your correfpondent, Dr. Kinglake, has done me the honour to pay me a compliment on the fcore of my humanity—I fay, with fomewhat lefs of the agreeable, becaufe, however dear the character of humanity may be to a medical man, there are, I believe, few in the profeffion who would thankfully accept of fuch a compliment, when coupled with an unneceffary reflection derogatory to his experience: that the reflection was unneceffary, and therefore impertinent, muft be evident to any perfon who recollects, that in my paper on the fubject of Digitalis, in your publication, I had *myfelf* difclaimed all pretenfions to experience in the cure of confumptions by means of that medicine; but it does not follow from this, that I am either inexperienced or timid in its adminiftration in other difeafes, or that I have expreffed any thing like a wifh to excite timidity in the regular practitioner. But, before I advance a claim to confiderable experience on this fubject, permit me to repeat my moft anxious wifhes, that medical men, when they are boafting of the virtues of this plant (which I admit to be ineftimable) will always have the humanity to guard the public at large againft the unwary ufe of fo infidious a poifon. The Digitalis is a plant which medical men may certainly ufe with fafety; but which they ought never to employ without much caution. I have already recorded one inftance of a lady preparing large and unlimited dofes for a friend in the laft ftage of a confumption; but I did not add, which I might then have done, that it would alfo have been adminiftered, with equal confidence, and in unlimited dofes, to two or three of her children, who were then afflicted with flight coughs.

In the year 1795, Mr. Martin, a refpectable publican, was taking the powder of Digitalis in the form of pills, by the prefcription of Dr. Lettfom:—while I was attending its adminiftration with all proper caution, I was one day ftruck with the appearance of a *large* bowl filled with the leaves of Digitalis infufed in boiling water, by his bedfide. Upon inquiry, I was informed that a paffenger in one of the ftage coaches, which had ftopped at his door, had told him that fox-glove tea was an infallible cure for the dropfy. I believe I need not add, that I refcued this patient from premature diffolution; for his was one of thofe cafes in which I had previoufly afcertained that Digitalis would make no ufeful impreffion.

In the year 1788, Mr. J. K.* a ftrong man, fomewhat more than 40 years of age, in the early ftage of a dropfy, was fuddenly and unexpectedly carried off with all the dreadful diftrefs and jactitation which an over dofe of Digitalis fometimes produces: his death was pretty generally afcribed to apoplexy, and was indeed truly apoplectic. I fufpected that this plant had been ufed; but the fact was moft refolutely denied. I have, however, fince learned that he really fell a facrifice to the unguarded ufe of fox-glove tea, recommended by a perfon unacquainted with its nature, and ignorant of phyfic.

In the year 1787 or 88, Mr. James P——, a refpectable tradefman, about the age of 50, was afflicted with a dropfy. The late Dr. Hugh Smith, and the prefent moft excellent Phyfician Dr. Wm. Sanders, prefcribed for him without alleviating his complaints. I propofed the Digitalis to each in turn, and each declined its ufe. It fell to my lot to adminifter it on my own judgement.—A few defert fpoonfuls of the decoction compleatly difcharged the water by urine, without exciting one unpleafant fymptom. The patient continued well and happy fome months, till a return of the diforder was gradually ufhered in by a return of cough and dyfpnoea. It was again removed in the fame pleafant and benign manner, by the fame quantity of the decoction of Digitalis. At the diftance of eight or nine months, the cough, dyfpnoea, and watery accumulation in the abdomen again appeared, and he had again recourfe to the Digitalis, which now produced not the fmalleft diuretic effect; unfortunately, the patient, eager to excite this evacuation, and full of confidence in a medicine which had been twice the means of preferving and prolonging his life, took a larger quantity than he had been directed to do, and, in the courfe of the night, with little or no previous notice, fell a fudden facrifice to its narcotic qualities.

Thefe

* For obvious reafons the initials are only printed, but the name at length is left with the Printer.

These are not all the cases in which I have had reason to suspect its furtive administration; and they may be considered, perhaps, as amounting to nothing more than the well-known consequences of an injudicious use of Digitalis. I grant they are so; but they, at the same time, afford the most convincing proofs, that, unless professional men will be much upon their guard, this valuable medicine, like laurel water, may do incalculable mischief: While *they* are administering it with all the caution of science and experience, in the form of pills, or powders, or tincture, the nurse, or the anile prescriber, will be pouring it down by cups full in the form of infusion or decoction.

I am glad that you afford room for the free discussion of this important subject, which is yet far from being exhausted; and I wish to add another page. It would, however, occupy too much of your excellent work, were I to enlarge upon the multiplicity of dropsical affections in which, during the last twelve years, I have administered this noble remedy with the most heart-felt satisfaction and success. I have the pleasure daily to see persons in good health, who, but for its use, would have been long since numbered with the dead.

——— Hawkins, a labouring man, upwards of 50 years of age, ten years since was relieved from a confirmed dropsy by the decoction of Digitalis. The disorder in twelve months returned, and was again permanently removed by the same remedy. He is now at the age of 60, or more, a labouring man, free from this disease.

——— Brunt, upwards of 70, was rescued from the most imminent danger, near twelve months since, by a few grains of the powder; and is now equally free from the disease.

——— Grey, a young woman, with confirmed dropsy, her abdomen equal in size to that of a woman at the full period of gestation, after the unsuccessful use of various other medicines, was speedily and effectually cured by the decoction of Digitalis, combined with the late Dr. Griffiths's chalybeate mixture, and the electuary of cream of tartar. She is now a hearty married woman, the mother of one or more children.

I could enumerate patients of almost every age, who have in my hands experienced the beneficial effects of Digitalis in various forms; and, during the course of an assiduous attention to these, I have been enabled to make the following general observations respecting its use, with which I will conclude this paper.

Whoever cultivates the Digitalis in his own garden, will, during six or eight months in the year, be able at all times to procure a decoction of the recent herb for extemporaneous prescription; fcription; and will perhaps find the decoction of the recent herb the beft and moft manageable preparation.

When fmall dofes of this decoction produce little or no effect, it will feldom be of much ufe, or fafe, to perfevere with large ones.

By one drachm added with the approbation of a very experienced phyfician (bis vel ter die) to one of his prefcriptions, a pair of anafarcous legs and thighs were in a few days reduced to their proper fhape; and a pair of lungs, labouring under ferous accumulation and fpafmodic oppreffion, freed during feveral months.

In drying the plant, the largeft, foundeft, and moft luxuriant leaves fhould be feparately laid on a table, and placed near the window of a green houfe, or any large room, expofed to the fun, that they may be quickly dried, and the green colour preferved.

If the leaves, when properly dried, be cut fmall like the advertifed Britifh herb tea or tobacco, and ftrongly compreffed into a canifter, or one of the drawers of an apothecary's fhop, and covered with writing paper, they will, in a dry fituation, preferve their virtues unimpaired for ten years; but it is much better to preferve a frefh quantity every year.

One ounce of the dried leaves is equal to four of the green, for the purpofe either of decoction or tincture. I have been, for many years, conftantly provided with a tincture drawn both with proof fpirit and nitrous æther, and have fometimes thought the latter the moft efficacious.

When the Digitalis is given in fubftance, the SEEDS are, on many accounts, preferable to the dried leaves.—In them the whole virtues of the plant are concentrated—they are exceedingly minute, and, in fact, a powder already prepared, and held out to us by the bountiful hand of Nature, requiring little or no care either in drying or preferving.

The moment they are bruifed in a mortar, the peculiar odour of the plant will be recognifed—combined with nitre, or cream of tartar, they form a convenient powder; and one grain will be found an active dofe.

Prefuming that their ufe is at prefent nearly confined to my own practice, and as it will be many months before they can be eafily obtained, if Dr. Kinglake fhould think it worth his while to adopt them on the ftrength of my EXPERIENCE and recommendation, I will moft cheerfully fupply him (as I now do you) with a fmall quantity.

It is worthy of remark, as fomewhat curious, that the peculiar virtues of the digitalis, hyofciamus, nicotiana, ftramonium, genifta, and feveral others of the narcotic clafs, fhould refide in the feeds, while thofe of the white poppy are not only perfectly free from every fedative or narcotic property, but very efculent and nutritious. This laft remark is not, I believe, equally true of the papaver erraticum; I fufpect the feeds of that plant to be poffeffed of an anodyne property worthy of future obfervation.

*Enfield, Feb.* 14, 1800. JOHN SHERWEN.

*P. S.* After what I have advanced in a former paper on the fubject of Digitalis, as applied to the cure of confumptions, it is incumbent upon me now to add, that I have lately had one cafe of incipient phthifis ftrongly marked (Eliz. Mathews) in which I have great reafon to believe that the tincture of Digitalis contributed much towards performing what I truft will continue to be a perfect cure; but as the cafe originated in chlorofis, and the preparations of myrrh, fteel and kali had been alfo had recourfe to, fome doubts may be entertained refpecting the effect of the tincture.

